# A Literature Review: The History of Psychological Impact of Illness amongst People with Leprosy (PwL) in Countries across the Globe

**DOI:** 10.1155/2021/5519608

**Published:** 2021-11-09

**Authors:** Pati Aji Achdiat, Eko Fuji Ariyanto, Michael Nobel Simanjuntak

**Affiliations:** ^1^Department of Dermatology and Venereology, Faculty of Medicine, Universitas Padjadjaran–Dr. Hasan Sadikin General Hospital, Bandung 40161, West Java, Indonesia; ^2^Division of Biochemistry and Molecular Biology, Department of Biomedical Sciences, Faculty of Medicine, Universitas Padjadjaran, Bandung 40161, West Java, Indonesia; ^3^Department of Medicine, Faculty of Medicine, Universitas Padjadjaran, Bandung 40161, West Java, Indonesia

## Abstract

**Background:**

Leprosy is a chronic infectious disease for which effective therapy has been long since invented. Thus, the morbidity has been decreased as technology has advanced, but the permanent disability has continuously generated stigma for centuries. The stigma causes the emergence of a poor psychological impact on people with leprosy (PwL). These impacts make new PwL reluctant to get appropriate therapy for their initial symptoms and are, thus, troublesome in accomplishing the goals of the leprosy elimination program. The aim of this review is to provide the history of psychological impact amongst PwL in countries across the globe.

**Methods:**

This is a literature review study. A keyword-based search was conducted in digital libraries. Articles reporting on PwL's psychology and related issues, such as quality of life, opportunity of building a marriage, and getting hired, were included. The data were presented based on a leprosy history timeline with cutoff points, namely, the invention of promin (1941) and multidrug treatment (1970).

**Results:**

In total, 38 studies were included in this review. These studies showed that PwL's knowledge towards leprosy has been increasing; nevertheless, their attitude is still lacking. The emotional response was described by various negative feelings that had persistently occurred. These poor psychological impacts were followed by poor treatment-seeking behavior and resulted in low quality of life.

**Conclusions:**

From year to year, the PwL's knowledge about leprosy has been getting better; nevertheless, their attitude towards the disease is still poor. The emotional response, social participation, and quality of life of PwL are persistently poor due to the persistent stigma.

## 1. Introduction

Leprosy, also known as Hansen's disease (HD), is a chronic infectious disease caused by *Mycobacterium leprae*. It mainly affects the skin, peripheral nerves, mucosal surface of the upper respiratory tract, and the eyes. If inadequately treated, it may cause permanent disability [1]. As a result, it may cause complex problems in many aspects of PwL's lives, socially, economically, and culturally [2, 3].

Along with the development of medical science, effective therapy for HD has been proven; thus, the morbidity of HD has been significantly decreased [3]. Since the discovery of *M*. *leprae* as the etiology of HD by Dr. Gerhard Henrik Armauer Hansen of Norway in 1873, numerous researchers have attempted to find an effective therapy for HD. In the early 20^th^ century, doctors all over the world treated PwL by injecting them with chaulmoogra nut oil, but its long-term efficacy was questionable. In 1941, promin, a sulfone drug, was introduced as a treatment for HD, but unfortunately, it required many painful injections. Then, in the 1950s, Dr. R. G. Cochrane pioneered dapsone as the treatment of choice for HD. It had worked effectively at first, but *M*. *leprae* began developing dapsone resistance. Finally, in the 1970s, the first successful multidrug treatment (MDT) regimen for HD was developed. Subsequently, in 1981, the WHO began recommending the use of MDT, a combination of dapsone, rifampicin, and clofazimine, for at least 6 months as the first-line treatment of HD [4].

Even though the MDT has successfully decreased the incidence of HD patients, the permanent disability due to HD continuously generates stigma in the society [5]. The stigma results in prolonged negative impacts on the PwL's entire life, such as loss of occupation, social ties and reputation, difficulty in finding a life partner, divorce, and discrimination [6–8]. Therefore, HD is often called as a social killer [9, 10]. The stigma due to HD is more troublesome than the disease itself [11, 12].

The stigma due to HD has occurred for centuries. In the 4^th^ century, the increment of HD incidence made PwL to be isolated in a distinct place called the leprosarium. The leprosarium in this period functioned like a prison. PwL were considered as the lowest level of society members, together with those with malnutrition, population crowdedness, and poor sanitation. Later on, in the medieval times, HD was connotated as a punishment from God due to PwL's wickedness and sin [13, 14]. Since the 11^th^ century, the sickness perceptions towards HD havegradually changed because many aristocrats acquired this disease, and assistance towards PwL became an obligation for Christians. In the 13^th^ century, leprosaria started to develop and give decent care to PwL, befitting a hospital [14, 15]. In the 20^th^ to 21^st^ century, discrimination due to the stigma of HD still continues. PwL were forced to leave their houses, and they were socially isolated. In 1953, in Japan, PwL were segregated into the leprosarium, where males were sterilized and females were forced to abort their fetuses. It happened due to the miscomprehension of doctors about HD transmission at that time. They believed that HD was a hereditary disease. Afterwards, the policy was written off in 1996 [16].

The impacts of stigma cause the emergence of poor psychological impacts on PwL. These self-perceptions make new PwL hide their symptoms and reluctant to get appropriate therapy at the onset of HD symptoms [1]. Thus, deformity and disability due to HD may appear. Some of the PwL decide to get treatment at health facilities far away, so they would not be recognized. All of these lead to the difficulty of eliminating HD completely [17] as reflected on the WHO's unachieved goals for HD in the WHO Global Leprosy Strategy 2011–2015. In this period, the incidence of grade 2 disability (G2D) due to HD was constant [18]. In the sequel, the WHO set new goals (Global Leprosy Strategy 2016–2020). Some of the goals are to eliminate G2D cases in children, decrease the incidence of G2D to 1 case/1,000,000 population, and abolish national policies that discriminate PwL [19].

In the interest of achieving goals of eliminating HD, acceptable (sufferers-centered) and effective interventions are required. Therefore, it is necessary to understand the viewpoints of PwL as well as their psychological impacts. Many studies have focused on figuring out the society's sickness perception towards HD, and there are limited studies on the psychological impacts of PwL.

Therefore, a study depicting those limited studies will be immensely useful. The aim of this review is to provide the history of psychological impact amongst PwL towards HD in countries across the globe.

## 2. Materials and Methods

### 2.1. Search Strategy

This is a study with a literature review approach. The authors carried out an electronic search in the National Library of Medicine (PubMed interface), Google Scholar, Elsevier, Public Library of Science (PLoS), and Semantic Scholar, using “Psychological impact of Leprosy” OR “Psychological impact of Hansen's Disease” OR “PWL Self-perception” OR “Impact of Leprosy Stigma” as keywords, without the year of publication and geographical restriction. The reference list of all identified documents was scrutinized with the aim of identifying additional potentially eligible studies.

### 2.2. Selection Criteria

All literature was assessed for eligibility by the authors. We assessed the title and abstract of each article identified in the search. Afterwards, the articles were selected by their eligibility according to the inclusion and exclusion criteria. The inclusion criteria of this study are original articles with the study aim of assessing PwL's psychological impact or psychological impact-related variables (knowledge, emotional perception, and quality of life) towards HD, a full article, and available in English or Indonesian. The exclusion criteria of this study are study with children as respondents (interviewee bias), textbook, and editorial article.

### 2.3. Data Extraction

Data were extracted from the included studies by the authors. The data extracted included authors, countries where the sample was collected, year of sample collection, number of samples, research tools, and the psychological impacts or psychological impact-related variables/life experiences of PwL towards HD (quality of life, opportunity to get a job, building a marriage, help-seeking behavior, and barriers to accessing health services). In some studies, the authors find it difficult to attain specific research tools used.

## 3. Results and Discussion

### 3.1. Study Selection and Sample Characteristics

**Figure 1:**
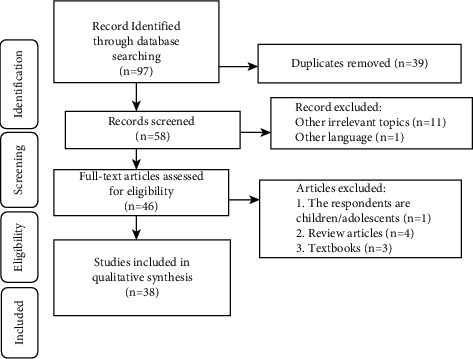
Study flow chart.

A flow of studies through the analysis is presented in Figure 1. Studies included in this review are listed in Table 1.

### 3.2. History of the Psychological Impact of PwL towards HD

#### 3.2.1. Period Subsequent to the Invention of Promin, the First Drug of HD (1941 to 1970)

From 1950 to 1953 in the United States, a study revealed that the prevalence of psychosis was significantly higher in PwL than in the general population, namely, about 10%. About 6.6% of PwL acquired schizophrenia. These were mainly caused by the thought that getting HD means an entire life of isolation from the community, family, and friends. Furthermore, some PwL often felt guilty and experienced a loss of self-esteem which culminated in depression [46]. In this period, denial frequently occurred in PwL. PwL were overwhelmed by the thought that they might have HD and refused to seek competent medical treatment for years until their extremities became bothersome. Even before being diagnosed, PwL reported that their family had refused to have close contact with them because their appearance was disturbing. Other PwL were reluctant to accompany their families in public and secluded themselves at home. These also might contribute to the occurrence of depression in PwL. Moreover, after being diagnosed with HD, many PwL admitted that they made an impulsive suicidal effort [20].

These findings showed that PwL's perception towards HD was poor in the period when only a few studies had been conducted on HD. Promin, as the first invented drug for HD, did not give effective results toall PwL, thus aggravating the disease severity, stigma towards HD, and PwL perception.

### 3.3. Period following the Invention of MDT for HD (1970 to Present) and PwL's Level of Knowledge towards HD

Some studies showed that the PwL still had low level of knowledge towards HD in the 1980s. In Thailand, in 1987, a study revealed that 26% of PwL believed karma as the cause of HD, while only 13% of them stated bacteria [21]. This finding is similar to a study conducted in the Philippines and Pakistan in 1988 and 1989, respectively. In Pakistan, PwL had a diverse psychological impact on HD. 47%, 20%, and 18% of PwL believed that mistake in diet, weather, and curse/magic were the causes of HD, respectively. Only 5.3% of PwL stated bacteria as the cause of HD [22]. In Nigeria, 58% of PwL believed foods, curses, taboos, and bad spirits were the causes of HD [26].

Studies conducted in Taiwan in 2003 and Nepal in 2004 showed that PwL were aware of the emergence of HD initial symptoms on their skin but they did not know the cause [28, 29]. These were similar with a study conducted in India in 2011 where most HD patients incorrectly knew their disease. About 75% did not know the cause of their illness. Others attributed it to contact with leprosy patients (7%), insect bites (3%), bacteria or viruses (12%), and alcoholism (3%). This study showed that there were still many PwL who had poor knowledge towards HD, despite having been diagnosed and given treatment [34].

In Indonesia, in 2013, some PwL thought that HD can easily spread by direct contact with PwL, breathing the same air, shaking hands, eating food prepared by PwL, and using the same personal objects used by PwL such as glasses, towels, and clothes. Moreover, a few PwL believed that having the same blood type, heredity, breastfeeding, swimming in dirty rivers, working with goats or cement, eating chicken, challenge from God, destiny, and sorcery can cause HD [10]. These findings are parallel to a study conducted in Brazil in 2014 which also revealed that most PwL were not able to explain the process of transmission, treatment, and cure of HD [37].

In India, in 2014, a study revealed that PwL had better knowledge of HD than the previous studies. About 91% of PwL knew correctly about the transmission modes of HD, 82% of PwL knew correctly about the duration of HD treatment, and 84% of PwL knew that HD is curable. However, their attitudes towards their disease were poor [38]. In Suriname, in 2016, a study showed that there were still many PwL who believed foods, family curses, and genetics as HD etiology. This belief was influenced by local culture, which forbids various foods to be eaten [41].

These studies showed that most PwL had poor knowledge towards HD in countries with the highest prevalence of HD cases, especially before 2013. After 2013, most studies revealed that PwL had a better level of knowledge regarding the etiology of HD but had still poor knowledge on its transmission. In 2014, 91% of PwL correctly knew about HD transmission. However, their attitude is still poor because HD visible symptoms are aesthetically disturbing. Even though the knowledge of PwL towards HD is generally good at present, a small number of PwL who live in areas with strong cultural value still believe in incorrect causes of HD. This finding is one of many factors contributing to the poor health-seeking behavior of PwL, thus making it difficult to stop HD transmission and its elimination.

#### 3.3.1. PWL Emotional Response towards HD

A study conducted in India in 1989 showed that PwL experienced a lot of burden due to the stigma of HD. About 82 PwL felt that others were afraid of them and hated them. They felt being underestimated and insulted. Moreover, 33% admitted that they had ever thought of attempting suicide [47]. In Thailand, in 1991, a study showed that PwL tend to avoid social participation and hide their symptoms [23]. These findings are similar to a study conducted in Brazil in 1997 which revealed that PwL felt that HD made them suffer and feel abandoned.

They felt guilty and ashamed because of their visible deformities. These lead to the emergence of social and emotional disorders [25].

According to studies conducted in the Netherlands and Taiwan in 2001, PwL felt that HD extremely burdened their lives. They avoid being active in the society so that others will not notice their symptoms [27]. Subsequent to being diagnosed with HD, PwL felt ashamed and stigmatized. Some of them thought that HD could not be eliminated from their lives even if the bacteria had been eliminated. This causes PwL to self-isolate themselves [29]. These findings are parallel to studies conducted in the Philippines and the Netherlands in 2010, which showed that PwL with visible disability had a significantly lower level of social participation [31, 32]. Moreover, studies conducted in Brazil in 2011 and Indonesia in 2012 and 2015 showed that PwL had lower social classes in the society, causing high percentages of secondary problems that negatively affect the work capacity and quality of life of PwL, thus perpetuating the stigma and ancient prejudice associated with the disease. Besides, it made PwL feel ashamed and decide to isolate themselves [10, 33, 48].

In Indonesia in 2013 and Brazil in 2014, studies showed that some PwL refer to their symptoms as “broken body.” Moreover, some PwL experienced HD reactions after being declared cured which made them continue to feel bad. Sadness, frustration, loss of confidence, devaluation of their own capacity, stress, and hopelessness were some emotions described by PwL [37, 40]. It is similar to a study conducted in Nepal in 2014, which showed that 59% of PwL had never told their family about their diagnosed disease due to low self-esteem, especially those with poor knowledge of HD [49].

In Suriname, in 2016, a study showed that PwL felt they were still ill even after they had been cured from HD because of the emergence of persistent stigma [41]. Moreover, a study conducted in Nigeria in 2016 revealed that PwL also experienced severe social participant restriction, especially those with G2D [42].

A study conducted in India in 2017 revealed that 79.5% of PwL felt afraid and worried about their disease. This occurred because of deformity and discrimination due to HD [43]. This is similar to a study conducted in Indonesia in 2019 which revealed that PwL felt shy and ashamed of their disease. They admitted that they regret seeking treatment because of the lack of knowledge of the initial symptoms of HD. The stigma felt was also personal, as well as the stigma they would receive from their family and surrounding environment [44].

These studies showed that, from year to year, PwL had persistent feelings towards their disease. Most of them felt sad, guilty, ashamed, stressed, hopeless, and frustrated and experienced low self-esteem. These feelings were caused either by the HD itself or the stigma and discrimination from their community, friends, and even family. How PwL feel about their disease emotionally also contributes to poor health-seeking behavior.

#### 3.3.2. PwL Access to Healthcare Facilities and Health-Seeking Behavior

Studies conducted in Pakistan in 1989 and Nepal in 2004 showed that 52% of PwL were uncompliant towards HD treatment and decided to get treatment from a traditional healer. This behavior occurred because of various reasons. Most PwL stated that they were disturbed by MDT side effects such as burning sensations, swelling, nausea, pain, and skin discoloration. These side effects were experienced by 57% of uncompliant PwL and 23% of compliant PwL [22]. This finding is similar to a study conducted in Nepal in 2004. Additionally, the stigma to the use of MDT blister packs and routine control by doctors also made PwL reluctant to be compliant [28]. Some PwL went to traditional healers and treated their lesions with oil [28], which is similar to a study conducted in India in 2013 [35].

Some PwL also said that they were not given adequate information regarding their disease from the doctors, while some others denied having HD and delayed seeking appropriate treatment for 3 years on average [22].

Studies conducted in Nigeria and Ethiopia in 1998 and 2000, respectively, showed that PwL tend to delay seeking treatment until the emergence of its complications. The delay was about 2 to 3 years after being diagnosed [26, 50]. This finding was also proven by a study conducted in Brazil in 2011. Additionally, this study also proved that the prevalence of more severe disability was higher in PwL who delayed seeking treatment longer [33].

A study conducted in India in 2011 showed that 15% of PwL did not seek treatment for HD because the patch remained unnoticed. The other 26% who had noticed their symptom(s) but did not seek treatment reported no experience of physical discomfort [34], whereas another study conducted in India in 2013 revealed a higher percentage, namely, 49.7%, believing the symptoms would disappear [35]. Other than that, about half of PwL did not continue the treatment for many reasons such as no visible effect of treatment (9%), high cost (18%), and distant location (5%) [34].

Other reasons attributed to the noncompliance of PwL were explained by a study conducted in Indonesia in 2013, namely, doctors' misdiagnosis of their conditions such as scabies, skin fungus, and sweat allergy. This causes PwL to not feel better and decide to return to the hospital, which might cause an economic burden [40]. An interesting study conducted in India in 2015 revealed that PwL had two most highly ranked issues regarding noncompliance towards HD treatment, namely, seeing improvement and not seeing improvement. PwL observe substantial improvement and, therefore, assume the treatment is no longer required, or conversely, discontinue treatment because they do not see any improvement and assume that the treatment was not effective [39].

These studies showed that PwL have diverse reasons for being uncompliant towards HD treatment, namely, the emergence of MDT side effects, not seeing improvement, seeing improvement, high cost, distant healthcare facility, not noticing initial symptoms, and underestimating initial symptoms' severity. Other than that, the stigma in the society towards the use of MDT blister packs and routine control by doctors increases the reluctance of PwL. The most unfortunate reason was doctors' misdiagnosis and inadequate information towards HD and its treatment.

On average, PwL delayed seeking treatment for 2 to 3 years after being diagnosed. The delay in seeking treatment for HD leads to a wider spread of bacteria invasion and worsens disease severity and, thus, leads to the emergence of complications such as deformity and disability.

### 3.4. The Opportunity of PwL to Get a Job and Build a Marriage

A study conducted in Brazil in 1997 showed that HD made PwL to have low desire to have sex not only from PwL themselves but also from their spouses. Besides, some PwL decided to resign from their job before their symptoms were noticed by their coworkers [25]. In Indonesia in 2012, a study revealed that PwL felt ashamed and it was difficult to find a life partner and get a job. Moreover, some of them were divorced, abandoned by their spouses, fired, and did not get paid [48]. This finding is similar to a study conducted in the same country in 2013 and 2015. PwL were avoided by clients because of fear of being infected by touching the objects or eating the food sold by PwL. PwL working as farmers are also faced with the stigma that people are afraid of eating rice from the fields planted by PwL because they stand in the same water during rice planting. Apart from the existence of the stigma of HD, this study also showed that PwL had difficulty in finding a job because they were not physically able to work [40].

The stigma that HD is a very infectious disease that can be transmitted by touching the same objects by PWL is worrisome. Besides, the impact of the stigma of HD can remain for a long time, even after being declared cured. These studies revealed that the stigma towards HD is the main reason for extreme social role restriction in PwL, thus leaving PwL with a persistent low quality of life.

### 3.5. PWL Quality of Life

A study in India in 1997 showed that PwL had lower scores of WHO Quality of Life (WHOQOL) in physical and psychological aspects, independency, social interaction, and environment [24]. Similar studies conducted in Bangladesh in 2003 and Brazil in 2012 revealed that PwL had a lower score of quality of life physically and psychologically than other society members. Physically, PwL experienced disability and pain. Psychologically, they were looked at negatively by the society due to their visible deformity [30, 51]. These findings were parallel to studies conducted in Ghana in 2013, India in 2017, and Indonesia in 2020 [36, 45, 52]. Moreover, fully recovered PwL, or those who had finished the MDT regimen, also had a low quality of life score [53].

These studies showed that the PwL's quality of life is persistently low from year to year, physically and psychologically. This was caused by the daily activity restriction due to physical deformities and social participation restriction due to the stigma in society, respectively.

## 4. Conclusions

From year to year, the PwL's knowledge about HD has been getting better; nevertheless, the attitude towards the disease is still poor. Other than that, their emotional response towards the disease is persistently poor. Procrastination of seeking treatment in PwL average between 2 and 3 years after being diagnosed. PwL admitted to having low quality of life and mental-related illnesses.

Many HD eradication programs have been held, namely, NLEP (National Leprosy Elimination Program) by the Indian Government and Leprosy Elimination Campaign (LEC) by the WHO [54, 55]. These programs consist of delivery of BCG vaccine and MDT chemoprophylaxis along with campaigns to increase PwL self-reporting and intensive information, education, and communication to reduce the stigma and discrimination against PwL. These interventions had given favorable impacts on reducing the prevalence of HD, but are still facing difficulty in achieving complete eradication due to undetected or hidden cases (1.90/10,000 population) [56]. In order to eliminate the incidence of HD and its complications, along with establishing a better life for PwL with disability, interventions are needed not only to reduce the stigma in society but also to improve knowledge and encourage PwL emotionally. Besides improving sustainable leprosy services, active surveillance as conducted in Leprosy Case Detection Campaigns (LCDCs) in India and China had greater impacts on reducing the prevalence of PwL, especially in an endemic area where hidden cases lead to continuous disease transmission [57, 58]. This kind of intervention should be implemented more stringently and considered in other endemic countries.

## Figures and Tables

**Table 1:** Articles included in the review.

No.	Author	Country	Year	Number of respondents/leprosy-affected persons (LAPs)	Research tools	Result
1	Lowinger	USA	1953	377	Complete psychiatric examination and psychiatric screening interview (not standardized)	Emotional response : PwL describe HD as an everlasting alienation from social life
2	Gussow	USA	1963	279	Intensive interview (not standardized)	Emotional response : PwL avoid showing up in public areas
3	Neylan et al.	Thailand	1987	61	Open-ended questionnaire adapted from Kleinman's explanatory model format 20	Knowledge : Only 13% of PwL stated bacteria as the etiology of HD, while most of them believe it is due to karma
4	Valencia et al.	The Philippines	1988	37	Questionnaire (standardized by the study researchers)	Knowledge : The level of knowledge of PwL towards HD transmission and etiology is poor Emotional response : Many PwL experience psychological stress
5	Chatterjee et al.	India	1989	17	General Health Questionnaire (GHQ) 21	Emotional response : PwL feel that stigma effects are extremely significant to their life
6	Mull et al.	Pakistan	1989	128	Questionnaire (standardized by the study researchers)	Knowledge : PwL have poor knowledge towards HD Health-seeking behavior: 52% of PwL are uncompliant towards MDT due to various reasons
7	Upayokin	Thailand	1991	25	In-depth unstructured interview (not standardized)	Knowledge : PwL have good knowledge towards HD Emotional response : PwL isolate themselves and refuse to participate socially. They experience psychological stress
8	Joseph and Rao	India	1997	50	WHOQOL22	Quality of life : PwL have lower QoL compared to the general population
9	Oliveira	Brazil	1997	202	Structured pretested questionnaire (standardized by the researchers) and in-depth structured interview (not standardized)	Emotional response: PwL feel that HD makes them suffer and are discriminated Building a marriage: PwL have low desire to have a sexual relationship Oppurtunity of employment: PwL decided to retire from their jobs to avoid stigma
10	Van De Weg et al.	Nigeria	1998	60	Questionnaire (not standardized)	Knowledge: PwL have poor knowledge towards HD Health-seeking behavior: On average, PwL delayed seeking treatment for 2 years after being diagnosed
11	Amenu et al.	Ethiopia	2000	79	Questionnaire (standardized by the researchers)	Health-seeking behavior: G2D PwL got treatment from traditional healers at the onset of HD symptoms. Appropriate treatment is delayed for 3 years on average
12	Steentjes	Netherlands	2001	150	In-depth interview (not standardized)	Emotional response: PwL feel that their disease is extremely severe and a burden on their lives. They refused to be promoted because it might expose their symptoms
13	Senturk and Sagduyu	Turkey	2001	65	Composite International Diagnostic Interview-Primary Health Care Version (CIDI-PHCV) 63, Brief Disability Questionnaire (BDQ) 64, and sociodemographic and clinical information form (not standardized)	Quality of life: PwL have a ratio of depression similar to that of the general population, but they have higher anxiety disorders
14	Heijnders	Nepal	2004	76	Interview (standardized by the researchers)	Knowledge: Most PwL lack knowledge about the initial symptoms of HD Health-seeking behavior: PwL felt that their disease was not severe initially, so they did not try to seek any treatment
15	Shieh et al.	Taiwan	2003	21	Focus group discussion/individual interview (not standardized)	Knowledge: PwL were aware of the emergence of HD symptoms, but they did not know the cause. Emotional response: After being diagnosed, PwL feel ashamed and stigmatized. This ruins their social life.
16	Tsutsumi et al.	Bangladesh	2003	189	WHOQOL-BREF22 and Self-Reporting Questionnaire (SRQ) 23	Quality of life: PwL have a lower QoL score compared to the general population significantly
17	Mullick et al.	Bangladesh	2008	100	Questionnaire (standardized by the researchers)	Quality of life: Most PwL experience major depressive illness, anxiety disorders, and dysthymic problems. Diagnosis of HD is the major factor causing these mental illnesses
18	Boku et al.	The Philippines	2010	70	Screening of Activity Limitation and Safety Awareness (SALSA) scale 24 and the General Self-Efficacy (GSE) scale 25	Emotional response: PwL with visible disabilities have a higher level of social participation than those without them
19	Slim et al.	The Netherlands	2010	82	WHO Disability Schedule II (DAS II) 68 and Impact on Participation and Autonomy (IPA) Questionnaire 26	Emotional response: 83% of PwL have extremity disorders. These disorders limit their daily activity and, thus, affect their social life
20	Lustosa et al.	Brazil	2011	107	SF-36 Questionnaire 27	Health-seeking behavior: The severity of disability parallels the period of treatment onset delay Emotional response: PWL with HD reactions are discriminated 4 times higher Quality of life It was found that leprosy still affects the lower social classes in historically endemic areas, causing high percentages of secondary injuries that negatively affect the work capacity and quality of life of affected people, thus perpetuating the stigma and ancient prejudice associated with the disease
21	Atre et al.	India	2011		Semistructured interview (not standardized)	Knowledge: Only 48% of patients correctly knew their disease, despite having been diagnosed and given treatment Health-seeking behavior: 24% of PwL delay seeking treatment for more than a year. 59% of PWL seek out traditional healers Barriers accessing healthcare services: 67% of PwL mentioned that they had not disclosed their condition to the community, suggesting an anticipation of stigma. The majority of patients (86%) mentioned that they had disclosed their condition to their family, and many (64%) acknowledged support from their spouse
22	Van Brakel et al.	Indonesia	2012	1358	Rapid disability appraisal toolkit: SALSA scale 24, participation scale28, Jacoby Stigma Scale (anticipated stigma) 29, Explanatory Model Interview Catalogue (EMIC) stigma scale 30, and discrimination assessment	Emotional response: Diverse problems are experienced by PwL due to disability. Stigma and discrimination from the society make PwL feel ashamed Marriage and employment oppurtunity: PwL find it difficult to have a spouse, build a happy marriage, and get hired. Some of them were abandoned, divorced, fired, and forced to work without being paid. These lead to a poverty cycle among PwL
23	Leite et al.	Brazil	2012	30	WHOQOL-BREF22	Quality of life: HD affects PwL's quality of life physically and psychologically
24	Peters et al.	Indonesia	2013	53	Interview (not standardized) and FGD (not standardized)	Knowledge: PwL have poor knowledge about HD transmission and etiology Barriers to accessing healthcare service: Some reasons that made PwL reluctant to seek medical treatment are that they did not have enough money and their primary-care location was far away. Some PwL underestimate the initial symptoms of HD Emotional response: PwL were complicated by the HD symptoms. They refer to their bodies as “broken.” Sadness, frustration, loss of confidence, devaluation of their own capacity, stress, and hopelessness were some of the emotions described due to leprosy. A few PwL said that they had considered ending their lives. Several PwL became reserved, shy, and ashamed and isolated themselves, but at the same time, several family members and people in the community also isolated those affected Employment opportunity: Some PwL were physically not able to do the work that they used to do. Some were fired because of leprosy, while others resigned themselves as suggested by family members. The impact can remain for a long time, even after being declared cured
25	Singh et al.	India	2013	245	Case studies and participant observation (not standardized)	Knowledge: Nearly half of PwL (49.7%) were unaware of the severity of symptoms such as red patches, minor swelling, and tingling sensations felt in wounds, believing that the symptoms would disappear Health-seeking behavior: The majority (26.93%) of PwL reported that, during the initial stages of their disease, self-medication such as massaging with mustard oil was sought for 4–5months
26	Bello et al.	Ghana	2013	70	HRQOL27	Quality of life: There was low QoL among the sampled elderly people affected by leprosy at the selected leprosaria (*p* < 0.05), thus stressing the need for measures that could improve their health and socioeconomic status within the settlements
27	Adhikari et al.	Nepal	2014	135	EMIC Questionnaire 30	Emotional response: 59% of PwL have never told their family about their disease. Better knowledge about HD made a better psychological impact
28	Loures et al.	Brazil	2014	20	Semistructured interview (not standardized)	Knowledge: Most PwL were not able to explain the process of transmission, treatment, and cure of HD. Emotional response: Most PwL reported negative feelings such as sadness, shame, and suffering
29	Rocha-Leite et al.	Brazil	2014	120	Mini International Neuropsychiatric Interview (MINI-Plus) 31	Quality of life: About 71.6% of PwL are diagnosed with having a mental illness
30	Stephen et al.	India	2014	100	Structured questionnaire (standardized by the researchers)	Knowledge: About 32% of PwL were aware that leprosy is due to infection caused by a germ A significant number of PwL had poor knowledge of the cause, mode of transmission, symptoms, referral pattern, cure, and prognosis of leprosy
31	Raju et al.	India	2015	320	Structured interview (not standardized)	Health-seeking behavior: Two of the most highly (and most consistently) ranked issues across respondents' type and region were seeing improvement and not seeing improvement. Discontinuing treatment because a person observes substantial improvement (and, therefore, assumes treatment is no longer required) or, conversely, discontinuing because the person does not see any improvement and assumes treatment is ineffective
32	Lusli et al.	Indonesia	2015	31	In-depth interviews (IDIs) and FGD (not standardized)	Emotional response: PwL shared feelings of being shy, sad, confused, afraid, and powerless in the face of the stigma and discrimination they faced from the outside world. They also talked about feelings of guilt and about hiding from others, by staying at home, for instance. Some voiced feelings of being a burden to their family. PwL explained that, since they believe what people say is true, they prefer to keep their feelings locked inside and not to share them with others PwL reported dealing with their own negative thoughts provoked either by what others think and say about them or by what they think about themselves. The participants affected by leprosy explained that the lack of social relationships could be due to their status as sick people Although the participants have said they consider themselves as part of their families and society, they simultaneously feel rejected by them due to their appearance: skin patches, physical deformity, and other visible impairments
33	Van Haaren et al.	Suriname	2016	13	B-IPQ32 and semistructured interview (not standardized)	Knowledge: PwL have poor knowledge towards the cause of HD Emotional response: PwL still have psychological impact and social and psychological disorders even after being cured
34	Ibikunle et al.	Nigeria	2016	63	EMIC, the Internalized Stigma of Mental Illness scale (ISMI) 33, participation scale 28, Eye, Hand, and Foot impairment (EHF) score 34, and the Social Distance Scale (SDS) 35	Emotional response: PwL have significant restrictions in participating socially, especially those with G2D
35	Govindharaj et al.	India	2017	358	Semistructured questionnaire (not standardized)	Emotional response: Most PwL (79.5%) feel afraid and worry about their disease due to deformity and discrimination
36	Govindharaj et al.	India	2017	100	WHOQOL-BREF22	Quality of life: PwL with G2D have lower QoL compared to those without it
37	Indow et al.	Indonesia	2019	6	In-depth interview (not standardized)	Emotional response: PwL undergo self-stigmatizing after being diagnosed with HD. Shyness and fear were expressed by PwL because they were late in reaching out for help or treatment. Reasons for respondents delaying their treatment were because they do not know the initial signs or symptoms of leprosy. The stigma felt was also personal, as well as the stigma they would receive from their family and surrounding environment, especially to informants who had experienced disability, because they would bear the shame of their lives because of their disability
38	Sinambela et al.	Indonesia	2020	30	WHOQOL22	Quality of life: Significant correlation perceived stigma and QOL
